# The influence of parenting style on health related behavior of children: findings from the ChiBS study

**DOI:** 10.1186/s12966-014-0095-y

**Published:** 2014-07-23

**Authors:** Nele Philips, Isabelle Sioen, Nathalie Michels, Ester Sleddens, Stefaan De Henauw

**Affiliations:** 1 Department of Public Health, Faculty of Medicine and Health Sciences, Ghent University, UZ 2 Blok A, De Pintelaan 185, Ghent, B-9000, Belgium; 2 FWO, Research Foundation Flanders, Brussels, Belgium; 3 Department of Health Promotion, NUTRIM School for Nutrition, Toxicology and Metabolism, Maastricht University Medical Center+, Maastricht, The Netherlands; 4 Department of Health Sciences, Vesalius, University College Ghent, Ghent, Belgium

**Keywords:** Parenting, Health, Soft drinks, Sedentary behavior, Sleep

## Abstract

**Objective:**

Exploring associations between parenting behavior and children’s health related behavior including physical activity, sedentary behavior, diet and sleep.

**Methods:**

We recruited 288 parents and their children (6-12y old). Children’s weight and height were measured. Fat percentage was determined by air displacement plethysmography. Parents reported socio-demographic data, sleep information, physical activity and sedentary behavior of their child and completed the Comprehensive General Parenting Questionnaire (CGPQ) and a Food Frequency Questionnaire. Children completed the Dutch Eating Behavior Questionnaire. Associations between parenting behavior (CGPQ) and children’s health related behavior were assessed with univariate and multiple regression analyses.

**Results:**

A small positive correlation was found between sweet food consumption frequency and “coercive control” (r = 0.139) and a small negative correlation between fruit and vegetables consumption frequency and “overprotection” (r = −0.151). Children consumed more frequently soft drinks when their parents scored lower on “structure” (r = −0.124) and higher on “overprotection” (r = 0.123); for the light soft drinks separately, a small positive correlation with “behavioral control” was found (r = 0.172). A small negative correlation was found between “emotional eating” and “structure” (r = −0.172) as well as “behavioral control” (r = −0.166). “Coercive control” was negatively correlated with the child’s sleep duration (r = −0.171). After correction for confounding factors, the following significant associations were found: (1) a small negative association between “structure” and soft drinks consumption (β = −0.17 for all soft drinks and −0.22 for light soft drinks), (2) a small positive association between “behavioral control” and light soft drinks (β = 0.34), (3) a small positive association of “nurturance” and “coercive control” with sedentary behavior (β = 0.16 for both parent constructs) and (4) a small negative association between the parenting construct “coercive control” and sleep duration (β = −0.23).

**Conclusion:**

The significant but small associations between parenting constructs and the investigated variables suggest that different aspects of parenting style play an important role in the genesis of the health related behavior of children. Overall, our findings suggest that health professionals should encourage parents to apply the more positive parenting constructs i.e., more “structure” and “behavioral control”, and less “coercive control”. They could, for instance, supervise and manage their child’s activities and help their child to achieve certain goals.

## Introduction

An estimated ten percent of the global population of school-aged children have excess body fat due to imbalance in energy homeostasis, putting them at an increased risk for developing chronic diseases later in life [1]. Indeed, several studies indicate that obesity-related behaviors of children track into adolescence and adulthood [2]–[4]. A better knowledge of the determinants of these behaviors in children would help to counteract these early pathways of chronic diseases. One possible determinant that is prominently in the picture nowadays is the influence of parents. More and more evidence is arising that children’s home environment can promote unhealthy dietary and exercise habits and thus promotes overweight; parents act as role models and largely control the availability and accessibility of food and possibilities for physical activity in children, influencing their children’s weight-related behaviors [5]–[9]. Moreover, parents also decide on the sleeping habits and sleep duration of young children, which is also known to be a determinant of overweight [10]–[12]. Besides these specific parental behaviors, the role of general parenting or “parenting style” is increasingly emphasized [13]. “Parenting styles” are a function of the parent’s attitudes, beliefs and behaviors, generating the emotional context for child. Several studies examined whether there is a link between parenting style and children’s health behaviors such as physical activity, eating behavior and risk taking behavior. The results of these studies are mixed. Jago et al. [7] and Hennessy et al. [14] found that parents with a permissive parenting style had the most physically active children. In contrast, Lohaus et al. [8] found that the authoritative parents had the children with the most positive health behaviors. In the context of a child’s body weight, Rhee et al. [15] demonstrated that children of authoritative mothers were significantly less likely to be overweight. Others also found an association between permissive parents and children with a higher Body Mass Index (BMI) [16],[17]. Sleddens et al. [13] found in their review that children raised in authoritative homes ate healthier, were more physically active, and had lower BMI scores compared to children who were raised with another parenting style. Nevertheless, some studies did not find an association at all [18]–[20]. A possible explanation for these different results could be the heterogeneity in definitions of parenting dimensions and corresponding behaviors. Furthermore, there is a considerable disagreement about how to best assess parenting. Most of the parenting questionnaires only assess limited aspects of parenting. In this study, we used the Comprehensive General Parenting Questionnaire (CGPQ), a recently developed instrument aimed to assess five key constructs of parenting (i.e., nurturance, structure, behavioral control, coercive control, and overprotection) [21]. This questionnaire measures the major individual dimensions in general parenting behavior, i.e. the strategies that parents use in child rearing, displayed across many different situations.

The overall goal of this study was to investigate the relationship between general parenting assessed with the CGPQ and the child’s weight related behaviors (i.e., diet, physical activity, sedentary behavior, and sleep behavior), and anthropometric outcomes (BMI and fat percentage). These health related behaviors were chosen because they will have an impact on the development of overweight. We hypothesize that general parenting will have an impact on the health related behavior of the children. We expect the more positive parenting behaviors (i.e., “nurturance”, “structure” and “behavioral control”) to be related to healthy behaviors and anthropometric outcomes (e.g., lower intake of sweets and soft drinks, higher level of physical activity, lower BMI and fat percentage). In contrast, we expect the more negative parenting behaviors (i.e., coercive control and overprotection) to be related to unhealthy behaviors and anthropometric outcomes (e.g., decreased sleep duration, higher BMI and fat percentage).

## Methods

### Study design and sampling

The children were recruited through random cluster design (all children from schools of the selected region Aalter) in the framework of the longitudinal Belgian ChiBS study (Children’s Body composition and Stress with measurements in 2010, 2011 and 2012) [22]. In this paper, only data from the cross-sectional survey conducted in 2012 was used, as we administered the CGPQ during this measurement period. Of the 455 children that participated in the ChiBS study in 2011, 331 participated again in 2012 (drop-out of 27.3%). Of the 331 participating children in 2012, only 288 children (87%) had complete data on the parenting constructs. Therefore, the study population for this paper includes 288 children. Based on the ChiBS data collected in 2011, we found that the 288 children participating in the analyses presented in this paper did not differ in age, gender, socio-economic status (SES) and BMI from the 455 children that participated in 2011 (p > 0.05).

The study was conducted according to the guidelines of the Declaration of Helsinki and was approved by the Ethical Committee of the Ghent University Hospital. All participants signed an informed consent.

### Measurements

#### Fat mass determination by air displacement plethysmography

To determine the body fat percentage, Air Displacement Plethysmography (ADP) was used (BODPOD®, Software version 4.2.4, Life Measurement Inc, Cranlea and Co, Birmingham, United Kingdom). ADP is considered as a good reference technique for body composition measurements with a quick, comfortable, automated, non-invasive and safe measurement process, making it feasible in children [23]. Children had to refrain from physical activity and food consumption two hours before the measurement. The device was calibrated daily according to the manufacturer’s guidelines. Children were measured twice in tight-fitting bathing suit with swim cap to rule out air trapped in clothes and hair. Thoracic gas volume was predicted by the software with a validated child-specific equation and fat mass percentage was calculated using the equation reported by Wells [23]. If the first and second measurement of the body volume differed more than 150 ml, a third measurement was performed. To obtain reliable and valid body composition measurements, the ADP technique was conducted in all children by one trained study nurse.

#### Routine anthropometry

The routine anthropometric measurements were carried out in all children by one trained study nurse. Children’s weight was measured in underwear with an electronic scale linked to the BodPod ®(Tanita Corporation, Japan (Model BWB-627-A), modified by Life Measurement, Inc.) to the nearest 0.1 kg and height was measured with a stadiometer (SECA 225) to the nearest 0.1 cm. BMI (kg/m^2^) was calculated (weight (kg)/height^2^ (m^2^)). Age- and gender- specific BMI z-scores were calculated according to Cole’s method [24]. BMI categories for children were determined using the International Obesity Taskforce (IOTF) BMI categories [25]. Parental weight and height were self-reported. For the parents, BMI categories were determined as recommended by the World Health Organization (underweight BMI < 18.5 kg/m^2^, overweight BMI > 25.0 kg/m^2^, obesity BMI > 30.0 kg/m^2^) [26].

### Questionnaires

#### Socio-demographic information

Parents reported their own educational level as well as that of their spouse and family characteristics (number of children, family structure, and number of older, younger or same-aged siblings than the participating child). Information on family structure was missing for 9 of the 288 children. Parental educational level was categorized according to the International Standard Classification of Education (ISCED): (level 0 ‘pre-primary education’, 1 ‘primary education’, 2 ‘lower secondary education’, 3 ‘upper secondary education’, 4 ‘post-secondary non-tertiary education’, 5 ‘first stage of tertiary education’, 6 ‘second stage of tertiary education’). The maximal ISCED level of both parents was used (this information was missing for 8 of the 288 children (2.8%) and based on information from only one parent for 13 of the 288 children (4.5%)). Most of the parents participating in this study had an educational level of 4 or more. Therefore, three groups were created: group 1 for the parents with educational level 0, 1, 2 or 3, group 2 for the parents with educational level 4 and group 3 for the parents with educational level 5 or 6. Children were described as youngest, oldest or middle child (when not being the youngest or the oldest).

#### Physical activity and sleep (parental-reported)

Parents were asked about the physical activity of their child: the hours of physical activity at sports clubs and outdoors per week was used. The number of screen time hours per week (i.e. television and computer time) was used as a measure of sedentary behavior. Parents also reported the typical hours of bedtime for weekdays and weekend days, from which the child’s average sleep duration per night was calculated as ‘(2*weekend + 5*week)/7’.

#### Comprehensive General Parenting Questionnaire (CGPQ) (parental-reported)

This 85-item questionnaire assesses five key parenting constructs that have been identified across multiple theoretical approaches of parenting: “nurturance”, “structure”, “behavioral control”, “coercive control”, and “overprotection” [21]. Each parenting constructs consists of different sub-constructs, each containing five items. It was designed by Sleddens et al. [21] and was validated in The Netherlands, Belgium and the United States. Parents were asked to indicate on a five-point Likert scale how much they agreed with each statement about parenting, ranging from 1 (*strongly disagree*) to 5 (*strongly agree*). The first construct ”nurturance” is the degree to which parents foster and recognize individuality and self-assertion by being supportive and responsive to their child’s needs, showing interest in child activities, spending time with their child, praising their child for good behavior, and expressing affection and care toward their child. It is composed by the parenting constructs ‘social rewarding’, ‘responsiveness’, ‘autonomy support’ and ‘involvement’. Parents scoring high on the second construct “overprotection” show higher scores on the two sub-constructs ‘excessive involvement’ (excessive nurturing) and ‘excessive monitoring’ (strict control). This negatively impacts child development through interfering with the development of children’s autonomy. The degree ‘excessive’ is applicable when parents show a level of involvement or monitoring that fits for a much younger child. The third construct “structure” indicates the degree to which parents organize their child’s environment, by helping their child when necessary to gradually achieve a certain goal, and consistently enforcing rules and boundaries. It consists of the sub-constructs ‘inconsistent discipline’, ‘consistency’, ‘organization’ and ‘scaffolding’. The fourth construct “behavioral control” can be regarded as parents’ supervision and management of their child’s activities, providing clear expectations for behavior and using disciplinary approaches in a non-intrusive manner. Parents scoring high on behavioral control provide adequate levels of control, they are not too strict or over-controlling, but rather allow their child to have enough space to develop independence and autonomy. It is composed of the parenting sub-constructs ‘monitoring’, ‘maturity demands’, ‘nonintrusive discipline’ and ‘considering child input’. The fifth construct “coercive control” is characterized by pressure, intrusion, domination, and discouragement of child independence and individuality. It consists of the parenting sub-constructs ‘psychological control’, ‘physical punishment’ and ‘authoritarian control’. The questionnaire was completed by the parent that accompanied the child to the survey center. In most cases, the mothers accompanied their children. However, we do not know exactly the number of questionnaires completed by mothers and/or fathers. Mean scores were calculated for each construct providing that at least 60% of the items for each subconstruct was completed. Cronbach’s alphas for each of the five higher order constructs were as follows: nurturance 0.74, overprotection 0.63, structure 0.53, behavioral control 0.33 and coercive control 0.63.

#### Children’s eating habits questionnaire (CEHQ)- food frequency questionnaire (FFQ) (parental-reported)

The CEHQ-FFQ is a screening instrument designed to investigate food consumption frequency and eating behaviors of children. It consists of 43 questions and was developed and validated within the EU FP7 IDEFICS project [27],[28]. Parents were asked to report on the frequency of their child’s consumption of selected food items in a typical week during the preceding 4 weeks using the following response options: ‘never/less than once a week’, ‘1-3 times a week’, ‘4-6 times a week’, ‘1 time per day’, ‘2 times per day’, ‘3 times per day’, ‘4 or more times per day’ or ‘I have no idea’. There were no questions about portion sizes. In this study we used the following categories: snacks (nuts, seeds, chips, popcorn, savory pastries, chocolate, candy, cookies, ice cream), sweet food (sweet drinks, sweet sandwich filling such as jam and chocolate spread, sweet breakfast cereals, sweeten diary and sweet snacks), soft drinks (light and non-light), fatty food (fried potatoes, high fat sandwich filling such as butter and chocolate spread, high fat dairy, sauces, cheese, fat meat preparations and high fat snacks), and finally the healthy group of fruit and vegetables. When parents did not fill in a question that was needed to calculate the consumption frequency of a certain food group, this lead to a missing value for that food group and that child.

#### Dutch eating behavior questionnaire (DEBQ) (child-reported)

The DEBQ is a 33-item questionnaire that assesses three types of eating behavior in children: eating in response to negative emotions (emotional eating, 13 items, e.g. *Do you have a desire to eat when you are emotionally upset?*), eating in response to the sight or smell of food (external eating, 10 items, e.g. *If you have something delicious to eat, do you eat it straight away?*) and eating less than desired to lose or maintain body weight (restraint eating, 10 items, e.g. *Do you try to eat less at mealtimes than you would like to eat?*). In all three types of eating behavior, the appropriate self-regulating mechanism of food intake is diminished or lost. Children could answer the questions with ‘never’, ‘almost never’, ‘sometimes’, ‘often’ or ‘very often’ as response alternatives [29]. Answers were than recoded in digits (never = 1, almost never = 2, sometimes = 3, often = 4 and very often = 5). When children did not fill in a question that was needed to calculate the score for one of the three eating behavior types, this lead to a missing value for the score of that eating behavior type. The following Cronbach's alpha values were found: emotional eating 0.91, external eating 0.77 and restrained eating 0.88.

### Statistical methods

The analyses were conducted using SPSS, version 21.0. The cut-off for significance was chosen at p < 0.05. Variables with a non-normal distribution were log-transformed. Descriptive statistics were performed to determine means and standard deviations for continuous variables and frequencies for categorical variables. Pearson’s correlations were used to test associations among continuous key variables. Based on the work of Cohen [30] we considered correlation coefficients smaller than 0.3 as small, those between 0.3 and 0.5 as moderate and those larger than 0.5 as large. ANOVA analyses and t-tests were used to test differences among key variables between groups. When a significant association was found between a parenting construct and a variable, the associations between the corresponding parenting sub-constructs and that variable were also tested for significance (e.g. when a significant association was found between “overprotection” and maternal BMI, than we also tested the relation between the maternal BMI and the sub-constructs of “overprotection” – being “excessive monitoring” and “excessive involvement” – were investigated. Multiple linear regressions were performed to determine the relationship between the parenting constructs and the key variables. First, the association of family characteristics (number of children, educational attainment of the parents, BMI of the parents) and child characteristics (age, gender, BMI of the child and child birth order in the family) with parenting behavior was investigated. Second, it was investigated whether the parenting behavior shows an association with several types of the children’s health related behavior (food consumption frequency, eating behavior, physical activity, sedentary behavior and sleep duration) controlling for child and parental characteristics. All models were corrected for the child's age and gender, parental ISCED-level, BMI father, BMI mother, child birth order and the other parenting constructs assessed by the CGPQ. The confounders were chosen on the basis of existing knowledge [6],[31] and univariate analyses done in this study, i.e. parameters that showed some evidence of association (p < 0.05). In model 1, the mutual relation between the different parenting constructs was tested. In five other models, the association between the parenting style and health related behaviors of the children were tested: in model 2 with the food consumption of the children, in model 3 with eating behavior, in model 4 with physical activity and sedentary behavior, in model 5 with sleep duration and in model 6 with children’s body composition (i.e., BMI z-scores and fat mass percentage). In model 6, we also adjusted for sleep duration, physical activity and consumption frequency of different food items: sweet foods, soft drinks, fruits and vegetables. This was done because these parameters are known to influence children’s body composition.

## Results

### Demographic characteristics of the study population

In total 288 parents (mainly mothers) and their children (145 boys and 143 girls) participated in this study. Demographic characteristics are presented in Table 1. Children were between 6 and 12 years old (mean 9.3 years, SD 1.51); 77.4% had a normal BMI for their age, 15.6% were underweight and 6.9% were overweight or obese. Most of the parents had a higher education. The majority of the mothers had a BMI in the normal range (69.0%), 26.8% was classified as overweight or obese. The percentage of normal-weight fathers (52.9%) was comparable to the percentage of fathers with overweight or obesity (47.1%). Most families had two children.

**Table 1:** Sociodemographic descriptives of the study population (n = 288)

	**%**	**N**
**Child characteristics**		
**Gender**		
Male	50.5	145
Female	49.5	143
**Age (years)**		
6	2.4	7
7	13.5	39
8	14.6	42
9	19.8	57
10	25.0	72
11	20.1	58
12	4.5	13
**BMI**^**$**^		
Underweight	15.6	45
Normal	77.4	223
Overweight/obesity	6.9	20
**Parental & family characteristics**		
Education*		
Primary	0	0
Lower secondary	1.1	3
Upper secondary	30.0	84
Post-secondary non-tertiary	13.9	39
First stage of tertiary	55.0	154
**Family structure**		
Traditional &	84.4	243
Non-traditional &	12.5	36
**Number of children**		
1	6.4	18
2	53.2	150
3	34.0	96
≥4	6.4	18
**BMI mother**^**$**^		
Underweight	4.2	12
Normal	69.0	196
Overweight/obesity	26.8	76
**BMI father**^**$**^		
Underweight	0	0
Normal	52.9	137
Overweight/obesity	47.1	122

*1 = primary education, 2 = lower secondary education, 3 = upper secondary education, 4 = post-secondary non-tertiary education, 5 = first stage of tertiary education.

$BMI children was calculated based on measured length and weight. BMI parents was calculated based on self-reported length and weight.

&Traditional family structure was defined as living together with the biological mother and father. If this was not the case, it was defined as non-traditional.

Most parents scored rather high on the parenting constructs of nurturance (mean 4.49, SD 0.34), structure (mean 4.12, SD 0.38) and behavioral control (mean 4.16, SD 0.41), while parents scored lower on the parenting constructs of overprotection (mean 2.96, SD 0.59) and coercive control (mean 2.29, SD 0.52). There were significant correlations between the parenting constructs mutually (see Table 2). Structure and nurturance showed a high positive correlation (r = 0.553), indicating that parents providing structure to their children were also likely to provide parental nurturance. A moderate correlation (r < 0.5) was found between behavior control and structure; nurturance and overprotection. A small negative correlation was found between coercive control and structure as well as nurturance (Table 2).

**Table 2:** Pearson correlation coefficients (R) between the five different parenting constructs assessed by the Comprehensive General Parenting Questionnaire (n = 288)

		**Structure**	**Behavioral control**	**Coercive control**	**Overprotection**
**Nurturance**	R	0.553	0.427	−0.159	0.175
	p-value	**<0.001**	**<0.001**	**0.005**	**0.002**
**Structure**	R		0.436	−0.219	0.145
	p-value		**<0.001**	**<0.001**	**0.011**
**Behavioral control**	R			0.288	0.415
	p-value			**<0.001**	**<0.001**
**Coercive control**	R				0.285
	p-value				**<0.001**

*R:* Pearson correlation coefficients.

### Parenting versus family characteristics

A significant positive but small correlation (r = 0.138) was found between the parenting construct “behavior control” and BMI of the mother, indicating a trend that mothers scoring higher on “behavior control” have a higher BMI (Table 3). To specify, (data not reported in table) small positive correlation coefficients were found between the parenting sub-constructs of “monitoring” (r = 0.130; P = 0.021) and “non-intrusive discipline” (r = 0.141; P = 0.012) with maternal BMI. Additionally, a positive but low correlation was present between maternal BMI and “overprotection” (r = 0.159) (both “excessive monitoring” (r = 0.121; P = 0.033) and “excessive involvement” (r = 0.156; P = 0.006)). We also tested whether the gender of the child moderated the relation BMI mother - behavioral control: the positive significant relation only remained for girls (r = 0.166; P = 0.036). Fathers did not score differently on the parenting constructs depending on BMI. Note, however, that most questionnaires were completed by the mothers.

**Table 3:** Pearson correlation coefficients (R) between the five different parenting constructs and characteristics of the child, the parents as well as health behavior of the child (n = 288)

	**Nurturance**		**Structure**		**Behavioral control**		**Coercive control**		**Overprotection**	
	**R**	**p**	**R**	**p**	**R**	**p**	**R**	**p**	**R**	**p**
**Family characteristics**										
**BMI mother (kg/m**^**2**^**)**	−0.019	0.732	0.056	0.29	**0.138**	**0.015**	−0.016	0.784	**0.159**	**0.050**
**BMI father(kg/m**^**2**^**)**	−0.07	0.238	−0.016	0.796	0.019	0.747	−0.027	0.652	0.076	0.205
**Child characteristics**										
**BMI child (z-score)**	−0.045	0.426	0.007	0.905	0.059	0.298	−0.039	0.491	0.062	0.271
**Fat percentage child (%)**	−0.072	0.203	−0.008	0.885	−0.047	0.403	−0.012	0.83	0.084	0.139
**Age (years)**	−0.006	0.92	0.074	0.2	0.026	0.642	0.045	0.425	−0.02	0.723
**Food consumption**										
**Frequency of snacks consumption (times/week)**	0.01	0.863	0.046	0.426	0.087	0.128	0.013	0.827	0.011	0.851
**Frequency of sweet food consumption (times/week)**	0.006	0.913	−0.046	0.422	0.075	0.187	**0.139**	**0.014**	0.055	0.336
**Frequency of fatty food consumption (times/week)**	−0.038	0.504	−0.013	0.821	0.067	0.238	0.082	0.151	−0.053	0.355
**Frequency of Fruit & vegetable consumption (times/week)**	−0.003	0.96	−0.067	0.257	−0.014	0.806	0.08	0.169	**−0.151**	**0.009**
**Frequency of fruit consumption (times/week)**	0.041	0.481	−0.04	0.492	0.017	0.771	0.084	0.142	−0.094	0.102
**Frequency of vegetables consumption (times/week)**	−0.065	0.258	−0.054	0.357	−0.043	0.454	0.026	0.657	**−0.149**	**0.009**
**Frequency of soft drinks consumption (times/week)**	−0.026	0.651	**−0.124**	**0.031**	0.019	0.741	0.096	0.093	**0.123**	**0.031**
**Frequency of light soft drinks consumption (times/week)**	0.036	0.528	−0.079	0.17	**0.172**	**0.002**	0.101	0.076	0.091	0.113
**Frequency of non-light soft drinks consumption (times/week)**	−0.055	0.338	−0.109	0.058	−0.083	0.144	0.059	0.303	0.101	0.076
**Emotional eating**	−0.059	0.300	**−0.172**	**0.003**	**−0.166**	**0.004**	0.014	0.81	−0.016	0.778
**External eating**	−0.073	0.561	**−0.109**	**0.029**	−0.036	0.307	0.078	0.388	−0.03	0.189
**Restraint eating**	−0.033	0.203	−0.126	0.059	−0.058	0.533	0.049	0.173	0.075	0.605
**Physical activity and sedentary behavior**										
**Physical activity (hours/week)**	0.027	0.645	0.047	0.423	−0.032	0.583	−0.031	0.598	0.048	0.416
**Sedentary behavior (hours/week)**	−0.005	0.929	−0.054	0.347	−0.027	0.636	0.057	0.317	0.002	0.978
**Sleep duration**										
**Sleep quantity (hours/night)**	−0.014	0.806	0.013	0.82	−0.034	0.545	**−0.171**	**0.002**	0.002	0.967

R: Pearson’s correlation coefficient; p: p-value; the correlations with a p-value <0.05 are indicated in bold.

No significant associations were found between the parenting constructs and the number of children in the family or with the family structure (living together with the biological mother and father was defined as a traditional family structure; if this was not the case, it was defined as non-traditional).

Parents with a higher educational level (level 5–6) provided more structure to their children than parents with a lower education (level 0–3) (P = 0.015; highest education mean 4.18 SD 0.37, lowest education mean 4.03 SD 0.40). To specify (data not reported in table), the parenting sub-constructs “inconsistent discipline” (P = 0.013; highest education mean 3.27 SD 0.91, lowest education mean 2.97 SD 0.83) and “organization” (P = 0.01; highest education mean 4.28 SD 0.49, lowest education mean 4.04 SD 0.41) received significantly higher scores among parents with the highest level of education compared to parents with the lowest level of education. At the same time, high educated parents were more overprotective than parents with a lower education (P = 0.005; highest education mean 2.97 SD 0.59, lowest education mean 2.83 SD 0.59). The other parenting constructs (“nurturance”, “behavioral control” and “coercive control”) did not differ according to the educational level of the parents. Moreover, there were no differences between larger families and smaller families.

### Parenting versus child characteristics

We found significant differences in the parenting constructs according to the gender of the child and the child birth order in the family. No significant correlations between the parenting constructs and the children’s body composition (BMI or fat percentage) or age were found (see Table 3).

Considering gender of the child participating in the study, we only found a significance difference on the parenting construct “behavioral control”; parents with a participating daughter scored lower on behavioral control compared to parents with a participating son (P = 0.008, for daughters: mean 4.10 SD 0.42, for sons: mean 4.22 SD 0.39). Parents with a participating daughter specifically scored lower on the parenting construct “maturity demands” (P = 0.042) and “non-intrusive discipline” (P = 0.002).

Parents scored different on the parenting construct “coercive control” when the child was the youngest in the family compared to when it was the middle child (P = 0.008; youngest mean 2.23 SD 0.49, middle mean 2.46 SD 0.57) and when the child was the middle child compared to when it was the oldest child in the family (P = 0.024; oldest mean 2.26 SD 0.51). Considering the parenting constructs with regard to child birth order, the only construct that differed significantly was the parenting construct “physical punishment”. Parents of whom the participating child was the oldest scored higher on this construct (P = 0.025; youngest child mean 1.26 SD 0.57, middle child mean 1.55 SD 0.96, oldest child mean 1.27 SD 0.60).

### Correlations between parenting style and child health behavior

Overall, significant but small correlations (<0.3) with at least one of the parenting constructs were found for emotional eating, external eating, sleep duration and consumption frequency of sweet foods, vegetables and soft drinks. No significant correlations were found for restraint eating, consumption of fatty foods or snacks, and physical activity nor sedentary behavior. These results are presented in Table 3.

There were significant correlations between parenting constructs and food consumption frequencies. First, the consumption frequency of sweet foods was positively correlated with the score on “coercive control” (r = 0.139; P = 0.014). The only significant parenting sub-construct was the “authoritarian control” (r = 0.166; P = 0.003). Second, we found a small negative correlation between the frequency of fruit and vegetables (F&G) consumption by the children and the parenting construct “overprotection” (r = −0.151; P = 0.009). The frequency of F&G consumption was correlated with both the constructs “excessive monitoring” (r = −0.141; P = 0.014) and “involvement” (r = −.116; P = 0.045). The children in the study population consumed more frequently soft drinks (non-light and light) when their parents scored lower on the parenting construct “structure” (r = 0.124; P = 0.031), especially on the parenting construct “inconsistent discipline” (r = −0.166; P = 0.004), and when their parents scored higher on “overprotection” (r = 0.123; P = 0.031), specifically on the parenting construct “excessive involvement” (r = 0.194; P = 0.001). When only considering the non-light soft drinks, we found no significant correlation with any of the parenting constructs. However, when considering the light soft drinks separately, there was a small positive correlation with the parenting construct “behavioral control” (r = 0.172; P = 0.002). Both sub-constructs of ”behavior control”, “non-intrusive discipline” (r = 0.171; P = 0.002) and “maturity demands” (r = 0.286; P < 0.001), were positively correlated with the consumption of light soft drinks. When considering boys and girls separately, this relation was only significant for the girls (r = 0.213; P = 0.007).

We found a small negative correlation between the parenting construct “structure” and “emotional eating” (r = −0.172; P = 0.003). The lower the parents scored on “structure” (especially the sub-constructs “consistency” (r = −0.167; P = 0.003) and “organization” (r = −0.134; P = 0.018)), the more the child eats in response to negative emotions. The same relationship was found between the parenting construct “behavioral control” and “emotional eating” (r = −0.166; P = 0.004). The correlation was only significant for the parenting sub-constructs “maturity demands” (r = −0.145; P = 0.010) and “monitoring” (r = −0.162; P = 0.004). After stratifying by gender, the relation between “behavioral control” and emotional eating remained only significant for girls (r = −0.168; P = 0.036). A limited association was found between the parenting construct “structure” and external eating of the child (r = −0.162; P = 0.029).

In this study, a small negative correlation between the parenting construct “coercive control” and the child’s average amount of sleep was found (r = −0.171; P = 0.002). Both the underlying parenting sub-constructs “psychological control” (r = −0.190; P = 0.001) and “authoritarian control” (r = −0.158; P = 0.005) showed a small negative correlation with the child’s sleep duration.

### Multiple linear regression

Next, the associations between different aspects of parenting style on the one hand and a child’s health behavior and a child’s body composition on the other hand were assessed in multiple linear regression analyses correcting for the confounding factors (child’s age and gender, BMI of the parents, educational level of the parents, child birth order and the other parenting constructs). Each parameter of a child’s health behavior and a child’s body composition was investigated in a separate model (Tables 4 and 5). A small significant negative association between parental “structure” and soft drinks consumption (both overall and non-light drinks; β = −0.17 and −0.22, respectively) was found after correction for confounders as well as a positive association between parental “behavioral control” and the consumption of light soft drinks (β = 0.34) (Table 4). Furthermore, there was a significant positive association between the parenting constructs of “nurturance” and “coercive control” and sedentary behavior (β = 0.16 and 0.16, respectively) after correction for confounders as well as a significant negative association between the parenting construct “coercive control” and sleep duration (β = −0.23). The size of the standardized regression coefficients were limited, the strongest association was found between “behavior control” and the consumption frequency of light soft drinks.

**Table 4:** Adjusted associations between the five different parenting constructs and children’s food consumption

	**Food consumption frequency (times per week)**								
	**Snacks**			**Sweet food**			**Fatty food**		
	**B (95% CI)**	**ß**	**p**	**B (95% CI)**	**ß**	**p**	**B (95% CI)**	**ß**	**p**
Structure	1.05 (-1.70; 3.79)	0.06	0.455	0.08 (-6.13; 6.29)	0.00	0.979	2.13 (-3.34; 7.59)	0.07	0.446
Behavioral control	2.14 (-0.50; 4.78)	0.14	0.113	0.13 (-5.85; 6.11)	0.00	0.966	2.47 (-2.80; 7.73)	0.08	0.359
Coercive control	0.10 (-1.67; 1.86)	0.01	0.914	3.30 (-0.69; 7.29)	0.13	0.106	1.56 (-1.95; 5.07)	0.07	0.385
Overprotection	-0.13 (1.70; 1.44)	-0.01	0.868	0.71 (-2.84; 4.25)	0.03	0.697	-2.17 (-5.29; 0.96)	-0.10	0.176
Nurturance	-0.34 (-3.26; 2.59)	-0.02	0.821	1.99 (-4.63; 8.62)	0.05	0.556	-2.82 (-8.65; 3.01)	-0.08	0.345
	**Fruits and vegetables**			**Vegetables**			**Fruits**		
	**B (95% CI)**	**ß**	**p**	**B (95% CI)**	**ß**	**p**	**B (95% CI)**	**ß**	**p**
Structure	1.05 (-1.70; 3.79)	0.06	0.455	0.08 (-6.13; 6.29)	0.00	0.979	2.13 (-3.34; 7.59)	0.07	0.446
Behavioral control	-1.01 (-3.96; 1.95)	-0.06	0.506	-0.13 (-1.45; 1.19)	-0.02	0.846	-0.62 (-2.98; 1.74)	-0.05	0.607
Coercive control	1.62 (-0.33; 3.56)	0.12	0.105	0.27 (-0.60; 1.15)	0.05	0.542	1.25 (-0.31; 2.81)	0.12	0.118
Overprotection	-1.21 (-2.93; 0.52)	-0.10	0.172	-0.30 (-1.08; 0.48)	-0.06	0.453	-0.99 (-2.38; 0.40)	-0.11	0.163
Nurturance	1.10 (-2.11; 4.31)	0.05	0.504	-0.29 (-1.75; 1.16)	-0.03	0.693	1.41 (-1.17; 3.99)	0.09	0.286
	**Total soft drinks**			**Non-light soft drinks**			**Light soft drinks**		
	**B (95% CI)**	**ß**	**p**	**B (95% CI)**	**ß**	**p**	**B (95% CI)**	**ß**	**p**
Structure	**-1.59 (-3.16; 0.03)**	**-0.17**	**0.047**	-0.57 (-1.82; 0.67)	-0.08	0.369	**-1.02 (-1.75; -0.29)**	**-0.22**	**0.007**
Behavioral control	0.73 (-0.78; 2.25)	0.08	0.342	-0.76 (-1.97; 0.45)	-0.11	0.218	**1.50 (0.79; 2.20)**	**0.34**	**<0.001**
Coercive control	0.35 (-0.66; 1.36)	0.05	0.498	0.39 (-0.42; 1.20)	0.07	0.344	-0.04 (-0.51; 0.43)	-0.01	0.866
Overprotection	0.42 (-0.47; 1.32)	0.07	0.355	0.35 (-0.36; 1.07)	0.07	0.333	0.07 (-0.35; 0.49)	0.02	0.746
Nurturance	1.44 (-0.23; 3.11)	0.14	0.092	1.03 (-0.30; 2.36)	0.12	0.133	0.41 (-0.37; 1.19)	0.08	0.301

Each parameter of food consumption was investigated in a separate model. All models were adjusted for the child’s age, child’s gender, parental ISCED-level, BMI father, BMI mother, child birth order and the other parenting constructs (n?=?288).

ß: standardized regression coefficient; p: p-value; the significant associations (p?<?0.05) are indicated in bold.

**Table 5:** Adjusted associations between the five different parenting constructs and children’s health behavior and body composition

**Eating behavior**										
	**Emotional eating**			**Restaint eating**			**External eating**			
	**B (95% CI)**	**β**	**p**	**B (95% CI)**	**β**	**p**	**B (95% CI)**		**β**	**p**
Structure	−0.07 (−0.34; 0.21)	−0.04	0.649	−0.14 (−0.46; 0.19)	−0.07	0.412	−0.10 (−0.39; 0.18)		−0.06	0.471
Behavioral control	−0.21 (−0.48; 0.06)	−0.14	0.128	0.07 (−0.24; 0.38)	0.04	0.676	−0.11 (−0.38; 0.17)		−0.07	0.450
Coercive control	0.05 (−0.14; 0.23)	0.04	0.626	0.05 (−0.16; 0.25)	0.04	0.655	0.04 (−0.14; 0.22)		0.03	0.678
Overprotection	−0.01 (−0.17; 0.15)	−0.01	0.937	−0.04 (−0.23; 0.14)	−0.04	0.640	0.05 (−0.11; 0.21)		0.05	0.532
Nurturance	0.09 (−0.21; 0.39)	0.05	0.561	−0.12 (−0.46; 0.22)	−0.06	0.492	0.11 (−0.19; 0.41)		0.06	0.467
**Physical activity, sedentary behavior and sleep duration**										
	**Physical activity (hours/week)**			**Sedentary behavior (hours/week)**			**Sleep duration (hours/week)**			
	**B (95% CI)**	**β**	**p**	**B (95% CI)**	**β**	**p**	**B (95% CI)**		**β**	**p**
Structure	1.44 (−1.18; 4.06)	0.09	0.282	−0.88 (−2.18; 0.42)	−0.11	0.185	0.09 (−0.11; 0.30)		0.06	0.384
Behavioral control	−2.46 (−4.92; 0.003)	−0.18	0.051	0.07 (−1.17; 1.30)	0.01	0.918	0.07 (−0.13; 0.27)		0.05	0.509
Coercive control	0.89 (−0.77; 2.55)	0.09	0.294	**0.88 (0.06; 1.70)**	**0.16**	**0.036**	**−0.23 (−0.37; −0.10)**		**−0.23**	**0.001**
Overprotection	−0.05 (−1.55; 1.45)	−0.01	0.945	−0.56 (−1.30; 0.17)	−0.11	0.135	0.04 (−0.08; 0.15)		0.04	0.562
Nurturance	1.24 (−1.69; 4.18)	0.07	0.408	**1.40 (0.03; 2.77)**	**0.16**	**0.047**	−0.21 (−0.43; 0.01)		−0.14	0.057
**Body composition of the child**^**a**^										
	**BMI z-score**			**Fat percentage (%)**						
	**B (95% CI)**	**β**	**p**	**B (95% CI)**	**β**	**p**				
Structure	−0.01 (−0.43; 0.41)	0.00	0.961	0.36 (−2.46; 3.18)	0.02	0.804				
Behavioral control	0.20 (−0.22; 0.61)	0.08	0.353	−0.66 (−3.46; 2.14)	−0.04	0.645				
Coercive control	−0.05 (−0.32; 0.22)	−0.03	0.733	0.27 (−1.55; 2.10)	0.02	0.769				
Overprotection	0.05 (−0.19; 0.29)	0.03	0.690	0.66 (−0.95; 2.27)	0.06	0.421				
Nurturance	−0.28 (−0.74; 0.19)	−0.09	0.241	−0.58 (−3.70; 2.54)	−0.03	0.716				

Each parameter of a child’s health behavior and a child’s body composition was investigated in a separate model. All models were adjusted for the child’s age, child’s gender, parental ISCED-level, BMI father, BMI mother, child birth order and the other parenting constructs (n = 288)

β: standardized regression coefficient; p: p-value; the significant associations (p < 0.05) are indicated in bold

^a^These models were also adjusted for sleep duration, physical activity and consumption frequency of different food items: sweet foods, soft drinks, fruits and vegetables.

## Discussion

This study examined the associations between parenting style and health related behavior of children. Overall, significant correlations were found between the parenting constructs and parental education, BMI of the mother, emotional eating, external eating, consumption frequency of sweet foods, vegetables and soft drinks and sleep duration. Nevertheless, most of the correlations between parental constructs and health related behavior of the children were low (r < 0.3), indicating that general parenting probably operates as a more distal predictor of childhood weight-related outcomes than more proximal behavior-specific parenting practices, e.g. feeding practices. In contrast with previous research, we did not classify the parenting style into one of the four broad categories (authoritarian, authoritative, permissive, and uninvolved or neglectful), but we described different aspects of the parenting style in more detail by using five key constructs for which a score was calculated. By using for each parent–child pair the scores for the five constructs as assessed by the CGPQ, we tried to respond to most of the criticism given on previous research in the parenting area, namely, the poorly described definition of the different parenting characteristics. Moreover, using the CGPQ made it possible to distinguish between “behavioral control”, “overprotection” and “coercive control”, which allowed us to differentiate the positive aspects of “control” from the more negative aspects. As an example, we found a positive correlation between the construct “coercive control” and the consumption frequency of sweet food whereas a negative correlation was found between the construct “behavior control” and emotional eating. Although the questionnaire that we used did not classify parents in one parenting style we can state that the authoritative parenting style corresponds with the parenting constructs “nurturance”, “behavioral control” and “structure”. In the western European population the authoritative parenting style is the most prevalent parenting style [7], which is in line with our study, in which most of the parents scored high on the aforementioned parenting constructs.

In this study, parental education was significantly associated with two parenting constructs, i.e. “overprotection” and “structure”. Higher educated parents tend to overprotect their children more and gave them more structure than lower educated parents. This is in line with the results of previous research showing that higher parental education is positively associated with more monitoring, control and restriction [6],[32]–[34]. No other studies were found that reported a correlation between parental education and providing structure to their children. A possible explanation for this association (parental education versus structure) is maybe the awareness of higher educated parents about the importance of structure in a child’s life and their consistency in acting predictable upon the child.

This study also indicated that mothers with a higher BMI were more controlling and overprotective towards their children. This is in contrast with the study of Wardle et al. [35] where obese mothers were less controlling than normal-weight mothers. It should however be noted that the study of Wardle et al. [35] only considered the aspect ‘control’ in the context of eating. Mothers with a higher BMI could be more controlling and overprotective in response to the fear that their children will have the same weight-related problems as themselves.

We did not find an association between the parenting constructs and the BMI nor body fat percentage of the children. The results in the existing literature on this subject are mixed. Some researchers found an association between the parenting style or some parenting constructs and the weight of the children [15],[36],[37], while others did not find an association [20],[38]. One possible explanation for this is the distal relation between parenting and BMI. There are many factors that influence this relation and we were not able to consider all these factors. Furthermore, the findings of some researchers indicate that the impact of general parenting on children’s weight status depends on characteristics of both the child and the parents [13].

Concerning eating behavior, the results indicated that the parenting constructs of “structure” and “behavioral control” were negatively correlated with emotional eating. This is in line with the results of previous research, where more monitoring and authoritative parenting induced less emotional eating [39],[40]. In this study, there was no significant association between parenting constructs and restraint eating. However, we found a small negative correlation between the parenting construct of “structure” and “external eating”. This can possibly be explained by the fact that parents scoring high on the parenting construct “structure,” provide their children rules and boundaries. If parents impose rules about when and what children can eat, children have less possibilities to eat following an external clue. No other studies investigating the relation between general parenting and external eating were found.

In this study there was no association between the parenting constructs and physical activity. Other studies have shown that parents can influence the activity of their child by their own activity and by logistic support. But most studies did not find an associations between physical activity or sedentary behavior and general parenting [41]–[43]. Next, we investigated whether there was a relation between parenting and the children’s food consumption. In the current literature mixed results have been reported for specific food related parenting practices and children’s eating behavior [34],[36],[44]. A possible explanation is the heterogeneity of the definition of the parenting practices. In our study, parents that scored higher on “coercive control” had children that ate more frequently sweet foods. When parents score high on “coercive control”, this may possibly induce a negative atmosphere in the family. This can cause more stress for the children, leading to higher cortisol levels. Recent research showed a positive interaction between high cortisol levels and the intake of sweet foods [45]. In contrast, the children of parents that scored high on “overprotection” ate less frequent fruit and vegetables. When we considered fruit and vegetables separately, the relation only remained for the vegetables. We did not find other studies that reported an association between excessive involvement and excessive monitoring and fruit and vegetables intake. A possible explanation for our finding is that, when parents excessively monitor their children while eating, they also put pressure on them to eat more. After all, pressure to eat is in most studies associated with fewer intakes of foods [9],[46]–[49]. The parenting construct “structure” was in this study negatively associated with the frequency of soft drink consumption (total and light), while the parenting construct “overprotection” was positively associated with the frequency of soft drink consumption. Two kinds of soft drinks were considered: light and non-light soft drinks. When we considered the two categories of soft drinks separately the light soft drinks showed a positive association with “behavioral control”. Most studies investigating the relationship between parenting and the consumption of soft drinks only included adolescents. Only a few studies included children of 12 years or younger. These studies found a positive relation between the permissive parenting style and soft drink consumption. “Structure” can be viewed as a parenting construct that is lacking in a permissive parenting style.

To our knowledge this is the first study that investigated the relation between general parenting and children’s sleep duration. We found a significant negative association between the parenting construct of “coercive control” and the child’s average sleep duration. We could not formulate a possible explanation for this result. Further investigation to confirm and explain this result is necessary.

After correcting for the confounding factors that we chose on the basis of the existing literature and our results, some relations were no longer significant. We found a significant negative association between the parenting constructs “behavioral control” and “structure” and the amount of light soft drinks a child consumes daily as well as a significant negative association between the total soft drinks (light and regular) and the parenting construct “structure”. An association that we did not find in the univariate analysis is the positive association between the parenting constructs “nurturance” and “coercive control” with sedentary behavior in the multivariate model. Furthermore, the association between sleep duration and “coercive control” remained.

### Study strengths and limitations

The current study has several strengths and limitations. By using the air displacement plethysmography technology, we could precisely measure the fat percentage of the children. Moreover, many different aspect of children’s health related behaviors were measured: dietary consumption, eating behavior, sleep duration, physical activity, sedentary behavior and body composition. This allowed us to look at many different relationships. Several significant associations were found, however, most of the correlations and regression coefficients were small, indicating that also other factors besides those investigated in this study play a role in the relation between the parenting style and the health behavior of the children, e.g. more proximal behavior-specific parenting practices such as feeding practices. More focused studies are needed to get further insight in the role of the parenting style.

As a result of this broad scope taking on board different parameters related to health behavior, we performed a vast number of statistical analyses to test many different hypotheses. Table 3 shows the results of 125 correlation analyses (5 parental constructs multiplied with 25 child characteristics). Using a p-value of 0.05, we have a high chance (99%) that we have observed a significant result just by chance (false positive). Applying a Bonferroni correction, we would need to use a p-value of 0.0004 (0.05/125); resulting in the fact that none of correlations described in Table 3 would be considered significant. However, the Bonferroni correction is known to be very conservative and increases the risk on false negative results (correlations considered to be non-significant while they are significant). Applying this correction would lead to a result in which some corrections that are significant are not considered like that. Because of the exploratory nature of this study, it was decided not to adjust for multiple testing. The main purpose of the paper was to find out whether correlations exist between the different parental constructs as measured by the newly developed CPGQ and children’s health behavior. Significant correlations between different parameters were found, indicating that it is relevant to use this instrument further on in investigations studying in depth the role of parenting style on children’s health behavior. Further investigations to confirm the results found in this study need to include studies with a longitudinal design.

Because the parenting constructs were only investigated in a cross-sectional setting we cannot be sure they are invariable. Two earlier studies investigated whether parenting style is stable. They both found that parenting style was stable for at least two years [38],[50]. Recently, the CGPQ was applied in a longitudinal design to investigate the moderating role of general parenting on the relationship between food parenting practices and children’s dietary behavior [51]. However, given the cross-sectional design of this study, we should be careful with interpreting causality. Next, the internal consistencies (Cronbach’s alphas) for the CGPQ were quite low – particularly for structure and behavioral control. This could be due to possible heterogeneity of these constructs in this sample. Furthermore, the questionnaires were mainly completed by mothers. However, there is no evidence that this has introduced any bias. Another limitation is the self-reported parental BMI, parenting behaviors, the self-reported food consumption frequency and the self-reported physical activity and sedentary behavior. Therefore, it is likely that the present study yielded underestimates of associations between scale scores of the CGPQ and child health outcomes. Indeed, as Shiely et al. [52] demonstrated in their study, people tend to underreport their weight. Moreover, when reporting parenting style, food consumption and physical activity, people can have the tendency to give social desirable answers. Finally, we cannot generalize these results to other groups since the study population was mostly middle or high social class. This is a study with a rather exploratory nature and more studies are needed to establish scientific evidence on the relation between parenting and children’s health related behavior. If these results are confirmed, parents should be advised to apply the more positive parenting construct “structure”, e.g. by supervising and managing its activities and “behavioral control”, e.g. by helping their child to achieve certain goals.

## Conclusion

We found moderate associations between the parenting and family characteristics (parental education and BMI of the mother), child characteristics (gender and child birth order), food consumption (sweet food, vegetables and soft drinks), eating behavior (emotional eating and external eating), and sleep duration. After correcting for confounding factors, there was a significant association between the parenting constructs and light soft drinks, sleep duration and sedentary behavior. Based on these exploratory results, we can consider the parenting constructs “overprotection” and “coercive control” as rather negative parenting constructs and “nurturance”, “structure” and “behavioral control” as rather positive parenting constructs influencing health related behavior of children. However, more in depth and longitudinal studies are needed to confirm these results. If these results can be confirmed, it would indicate that public health programs should also focus on guidance of parents for developing more adequate parenting skills – in particular aiming at avoidance of “overprotective” and excessive “coercive control” and at promotion of more family structure and behavioral control of children.

## Competing interests

The authors declare that they have no competing interests.

## Authors’ contribution

NP did the statistical analyses, interpretation of the results and wrote the paper. IS and NM completed the fieldwork for the study and helped in performing the statistical analyses. IS revised the paper based on the comments of the reviewers and editor. ES designed and validated the CGP-questionnaire, helped in comparing the results with other study results. SDH coordinated the study and the field work. IS, NM, ES and SDH helped in interpreting the results of the statistical analysis and revised draft versions of the paper. All authors read and approved the final manuscript.
